# A Review: Laser Interference Lithography for Diffraction Gratings and Their Applications in Encoders and Spectrometers

**DOI:** 10.3390/s24206617

**Published:** 2024-10-14

**Authors:** Linbin Luo, Shuonan Shan, Xinghui Li

**Affiliations:** 1 Tsinghua Shenzhen International Graduate School, Tsinghua University, Shenzhen 518055, China; luolb24@mails.tsinghua.edu.cn (L.L.); shansn24@mails.tsinghua.edu.cn (S.S.); 2 Tsinghua-Berkeley Shenzhen Institute, Tsinghua University, Shenzhen 518055, China

**Keywords:** gratings, laser interference lithography, grating interferometry, encoders, grating spectrometer

## Abstract

The unique diffractive properties of gratings have made them essential in a wide range of applications, including spectral analysis, precision measurement, optical data storage, laser technology, and biomedical imaging. With advancements in micro- and nanotechnologies, the demand for more precise and efficient grating fabrication has increased. This review discusses the latest advancements in grating manufacturing techniques, particularly highlighting laser interference lithography, which excels in sub-beam generation through wavefront and amplitude division. Techniques such as Lloyd’s mirror configurations produce stable interference fringe fields for grating patterning in a single exposure. Orthogonal and non-orthogonal, two-axis Lloyd’s mirror interferometers have advanced the fabrication of two-dimensional gratings and large-area gratings, respectively, while laser interference combined with concave lenses enables the creation of concave gratings. Grating interferometry, utilizing optical interference principles, allows for highly precise measurements of minute displacements at the nanometer to sub-nanometer scale. This review also examines the application of grating interferometry in high-precision, absolute, and multi-degree-of-freedom measurement systems. Progress in grating fabrication has significantly advanced spectrometer technology, with integrated structures such as concave gratings, Fresnel gratings, and grating–microlens arrays driving the miniaturization of spectrometers and expanding their use in compact analytical instruments.

## 1. Introduction

**Figure 1:**
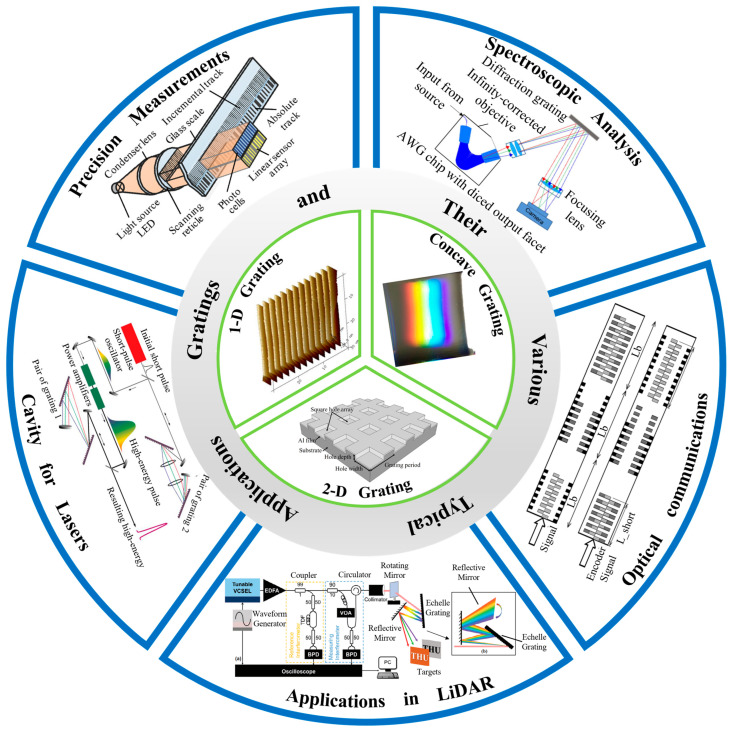
Gratings and their various typical applications [26,70,71,72,73,74,75,76].

Gratings are indispensable components in precision optics [1,2,3], playing a pivotal role across various scientific and industrial domains [4,5,6], including spectral analysis [7,8,9,10,11], precision measurements [12,13,14,15], cavity for lasers [16,17,18,19,20], optical communication [21,22,23,24,25], and LiDAR [26,27,28,29,30,31], as Figure 1 shows. Their unique diffractive properties are integral to high-precision applications, particularly in grating-based measurement systems and spectral analysis [32,33,34,35,36,37,38]. The grating period, typically ranging from 0.5 to 1.5 μm [39,40,41,42], is critical in determining the precision of instruments such as spectrometers and grating scales [43,44,45,46,47,48,49,50].

To address the challenges associated with fabricating gratings within this precise period range, researchers worldwide have pioneered numerous innovative techniques. These advancements have not only enhanced fabrication efficiency but also reduced costs and achieved highly uniform grating structures [51,52,53,54,55,56,57]. The spectrum of grating fabrication methods includes laser interference lithography, mechanical ruling [58,59], optical projection lithography [60,61,62], nanoimprint lithography [63,64,65], and electron beam lithography [66]. Table 1 describes the main micro- and nanofabrication technology characteristics of several commonly used fabricable gratings. Among these, laser interference lithography (LIL), also referred to as holographic lithography, distinguishes itself as a maskless technique based on coherent light interference [43,67,68,69]. LIL is favored for its high fabrication efficiency, precise period control, and system simplicity, making it a widely adopted method in surface science and nanoscience.

The types of gratings utilized in various instruments primarily include planar one-dimensional gratings and concave gratings, with two-dimensional gratings predominantly employed in planar and surface encoders. This review provides an in-depth exploration of the recent advancements in grating fabrication technologies, emphasizing their applications in grating-based measurement systems and spectral analysis. Compared to the existing reviews, this paper presents a more comprehensive introduction to grating processing methods utilizing LIL, as well as grating, in applications. The review details key techniques for fabricating planar and concave gratings using LIL. Following an introduction to the fundamental principles of LIL, the discussion expands on two primary beam-splitting systems: amplitude division and wavefront division. Additionally, the paper delves into several multi-beam interferometric lithography systems, with a particular focus on the orthogonal and non-orthogonal, two-axis Lloyd’s mirror interferometers. 

Further, the review highlights ongoing research efforts aimed at adapting these techniques for large-area grating fabrication, as well as strategies to mitigate phase drift during the exposure process. These advancements are crucial for enhancing the overall precision, reliability, and scalability of grating fabrication, which are essential for the continuous evolution of high-precision optical instruments.

Overall, the main components of this paper consist of five sections. Section 1: Introduction; Section 2: key developments in grating fabrication. The main content includes the principle of LIL (Section 2.1), 1-D grating fabrication (Section 2.2), 2-D grating fabrication(Section 2.3), concave grating fabrication (Section 2.4), and exposure field drift control (Section 2.5); Section 3: key developments in grating interferometry. The main content includes high-precision measurement (Section 3.1), absolute measurement (Section 3.2), multi-degree-of-freedom measurement (Section 3.3), and industrialization modules of grating interferometry (Section 3.4); Section 4: gratings in miniature spectrometers. The main content includes the concave grating spectrometer (Section 4.1), the Fresnel grating spectrometer (Section 4.2), and the novel microlens grating spectrometer (Section 4.3); Section 5: conclusion and prospects.

## 2. Key Developments in Grating Fabrication

**Figure 2:**
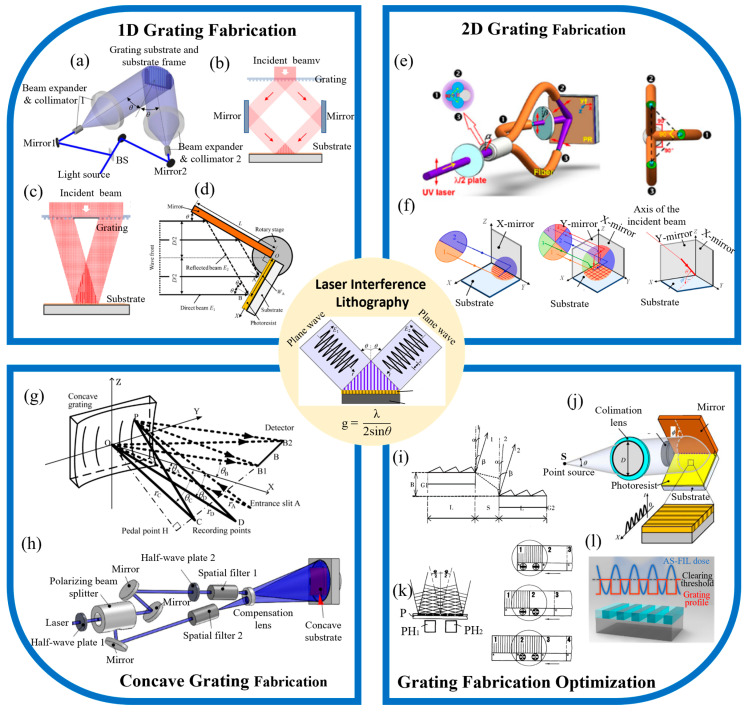
Key techniques in grating fabrication using LIL: (**a**) the amplitude division method for 1-D grating fabrication [89]; (**b**) an amplitude division system utilizes a transparent grating to generate positive and negative first-order diffracted beams [90]; (**c**) a schematic diagram of a two-beam direct interference system [91]; (**d**) a two-beam interferometric system realized by a single-axis Lloyd’s mirror interferometer [92]; (**e**) a multi-beam interference lithography system with a fiber bundle [93]; (**f**) the construction of a two-axis Lloyd’s mirror [94]; (**g**) a concave grating type spectrometer [95]; (**h**) a schematic diagram of a concave grating fabrication system [76]; (**i**) a schematic diagram of mechanical stitching [96]; (**j**) a system to replace a conventional laser source with a 405 nm laser diode for one-dimensional grating fabrication [92]; (**k**) a schematic diagram of exposure stitching [97]; (**l**) a schematic of grating with uniform duty cycle exposure by two flat-top beams [98].

Laser interference lithography (LIL) is employed to fabricate various types of gratings, including planar 1-D, 2-D, and concave gratings. Each type exhibits distinct periodic characteristics: 1-D gratings feature unidirectional periodicity [77], 2-D gratings have two-dimensional periodic variations [78,79,80], and concave gratings incorporate 1-D periodicity on a curved substrate [81,82,83]. The LIL process leverages the principles of laser interference to precisely control the grating parameters [77,84,85,86,87,88], thereby achieving specific diffraction outcomes. Some of the key techniques in grating fabrication using LIL are given in Figure 2, including the basic principle of LIL, 1-D and 2-D grating fabrication, concave grating fabrication, and parameter control techniques during interferometric lithography.

### 2.1. Principle of LIL

In dual-beam laser interference lithography, two coherent beams of identical wavelength are superimposed, creating an interference pattern on a photoresist-coated substrate. The visibility of this pattern is optimized when the beams maintain equal intensity and high coherence, which can be achieved by using a spatial filter. The period of the interference fringes, g, which is critical for grating fabrication, is defined by Equation (1):

(1) $$g=\frac{\lambda }{2\sin\theta }$$

where λ represents the wavelength, and θ is the incidence angle, as shown in Figure 2. The coherence length Lc, essential for maintaining pattern fidelity, depends on the laser’s linewidth Δλ and is calculated using Equation (2):

(2) $$L_{c}=\frac{\lambda^{2}}{\Delta \lambda }$$

These parameters are pivotal in ensuring the accuracy and quality of the fabricated gratings, allowing for precise control over the resulting diffraction properties.

### 2.2. 1-D Grating Fabrication

Planar gratings are effectively fabricated using LIL through a dual-beam interference process, producing the periodic line patterns characteristic of one-dimensional gratings. In this technique, the primary laser beam is split into subsidiary beams to maintain coherence. These subsidiary beams are generated using two main approaches: amplitude division and wavefront division.

The amplitude division method [89,99,100,101,102,103,104], as illustrated in Figure 2a, involves splitting the primary laser beam into two subsidiary beams via a beam splitter, which are then directed by mirrors to create interference fringes on the substrate [89]. This configuration offers significant flexibility in optical arrangement, allowing for the fabrication of large diffraction gratings by expanding the subsidiary beams. However, the extended non-common optical paths increase the susceptibility to external disturbances, necessitating active phase stabilization, which adds complexity to the setup. Another amplitude division system, shown in Figure 2b, utilizes a transparent grating to generate positive and negative first-order diffracted beams [90]. These beams superimpose to form a linear interference pattern, with the zero-order beam blocked by an aperture. Although this configuration slightly limits optical flexibility, it enhances stability by reducing the length of the non-common optical paths.

Alternatively, the wavefront division method [90,91,105,106,107,108,109,110], depicted in Figure 2c, generates positive and negative first-order beams from different regions of the same transparent grating, which are then superimposed on the substrate to form interference fringes [91]. The single-axis Lloyd’s mirror interferometer, shown in Figure 2d, represents another wavefront division approach [92]. It features a base and a perpendicular reflector, with part of the primary beam directly striking the substrate while the reflected portion is redirected on to the substrate by the mirror. This compact system minimizes the non-common optical path, leading to a more stable pattern exposure compared to amplitude division-based setups. Furthermore, Equation (2) illustrates that a narrow linewidth laser with a smaller Δ*λ* is essential for achieving a longer coherence length. An alternative approach involves using a low-cost laser with a broader linewidth to produce high-contrast interference fringe patterns. Traditional lithography systems typically rely on gas lasers, such as He-Cd lasers, which, while effective, are both expensive and bulky. To overcome these limitations, Li et al. developed a more cost-effective system by substituting the traditional laser source with a 405 nm laser diode for 1-D grating fabrication [92]. They further enhanced the diode’s coherence length by incorporating an external cavity, successfully fabricating a 1-D grating with a 570 nm period. This innovative approach offers a compact and economical alternative to conventional light sources, significantly reducing the system’s overall footprint.

On the other hand, the duty cycle, a key parameter of the grating profile, determines the diffraction characteristics of the grating [111,112]. Therefore, the uniformity control of the duty cycle is also crucial, prompting Xue et al. to develop an amplitude-split, flat-top beam interference lithography fabrication technique to improve the duty cycle uniformity [98], as shown in Figure 2l. The study analyzed the relationship between the duty cycle uniformity of the exposed beam and irradiance, and the results showed that the grating duty cycle inhomogeneity was kept below ±2% when the beam irradiance inhomogeneity was less than 20%. In addition, an experimental split-amplitude, flat-top beam interferometric lithography system was developed, which achieved a 21% irradiance inhomogeneity of the incident beam. The full aperture duty cycle inhomogeneity of the fabricated gratings was less than ±3%. Split-aperture, flat-top beam interferometric lithography improves duty cycle uniformity, greatly reduces energy loss compared to conventional traces, and is more suitable for fabricating large-area, highly uniform gratings.

### 2.3. 2-D Grating Fabrication

Two-dimensional gratings can be fabricated using either double-exposure or single-exposure methods [113]. The double-exposure technique involves rotating the substrate by 90° after the initial exposure using a one-dimensional grating setup, thereby creating an orthogonal two-dimensional grating structure. While this method facilitates the fabrication of sub-micrometer period gratings, it is prone to inconsistencies caused by environmental variations, timing discrepancies, and mechanical rotation errors between exposures. These factors can compromise the uniformity of the grating pattern, leading to varying groove depths and differences in diffraction efficiency. To overcome these challenges, single-exposure techniques that generate orthogonal two-dimensional interference serve as an alternative with higher precision.

**Figure 3:**
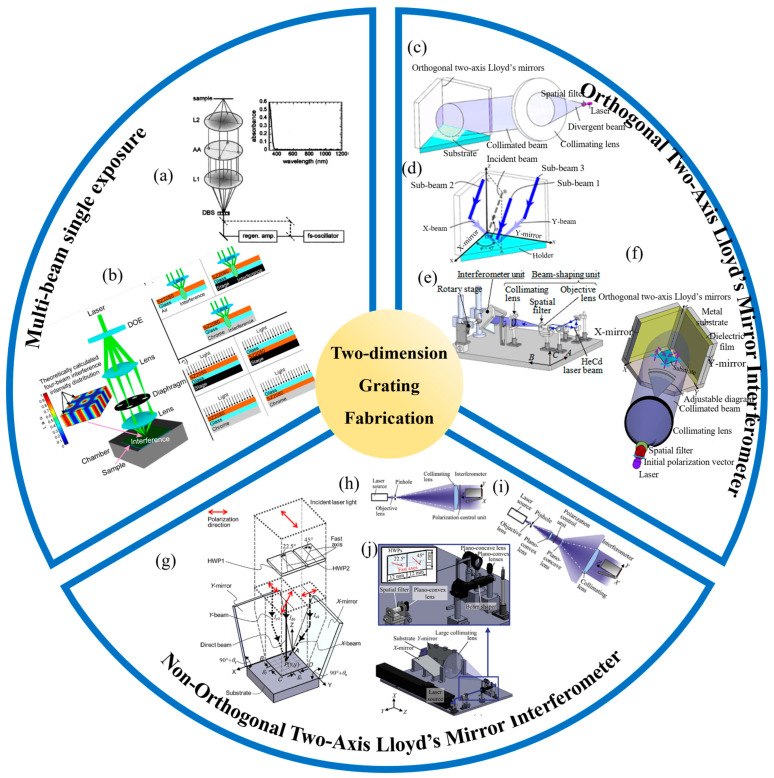
Key techniques for 2-D grating fabrication: (**a**) a schematic diagram of multi-beam interference using DBS [114]; (**b**) a pillar formation mechanism in four-beam interference lithography [116]; (**c**) a schematic diagram of an orthogonal two-axis Lloyd’s mirror interferometer system [124]; (**d**) the modeling of beam propagation in an orthogonal two-axis Lloyd’s mirrors interferometer [124]; (**e**) an orthogonal two-axis Lloyd’s mirror interferometer system with polarization modulation [125]; (**f**) a large-area, orthogonal two-axis Lloyd’s mirror interferometer system with polarization modulation [40]; (**g**) the modeling of beam propagation in non-orthogonal, two-axis Lloyd’s mirrors interferometer [126]; (**h**) beam expansion units for the fabrication of a large-area scale grating based on the Keplerian beam expander [126]; (**i**) beam expansion units for the fabrication of a large-area scale grating based on the Galilean beam expander [126]; (**j**) a schematic diagram of a non-orthogonal, two-axis Lloyd’s mirror interferometer for the fabrication of a large-area, two-dimensional scale grating [127].

In multi-beam interferometric lithography, where more than three subsidiary beams are used, all the beams are typically derived from a single primary laser source. An example of this approach is shown in Figure 3a, where a diffractive beam splitter (DBS) creates multiple beams, and an aperture array selects specific beams to form the interference fringe pattern on the substrate [114]. This configuration, which employs diffractive optical elements (DOEs) for beam generation, enhances the alignment and precision of the subsidiary beams [115,116,117]. Notably, Chua and Murukeshan from Nanyang Technological University have developed a single-exposure system using amplitude-splitting interference [115]. In this setup, circularly polarized light is diffracted by a two-dimensional transmission grating, with the zero-order light blocked. The resulting first-order diffracted beams in both X and Y directions are reflected onto the substrate, where they interfere to form the grating pattern in the photoresist. Further, Stankevičius et al. have explored the pillar formation mechanism in four-beam interference lithography [116], as illustrated in Figure 3b. They discovered that heat flow during photopolymerization influences the pillar base’s widening, highlighting the suitability of ultra-short pulse lasers for high-precision micro/nanofabrication. This insight is crucial for optimizing photopolymer materials and enhancing grating fabrication accuracy. Multi-beam interferometric lithography systems utilizing wavefront division have also been extensively documented [93,118,119,120,121,122,123]. Solak et al. demonstrated the creation of an orthogonal, two-dimensional interference pattern using four diffracted beams from a primary laser source, though the spatial extent of the interference field was limited by the grating pattern array’s size. To address this, methodologies like mosaic lithography have been proposed for fabricating large-scale two-dimensional gratings. Figure 2e shows a three-beam interferometric lithography system integrated with optical fibers [93], which simplifies angle adjustment and polarization modulation, thereby improving precision and versatility.

While these configurations are not ideal for mass production, the two-axis Lloyd’s mirror structure, initially developed by Zeng Lijiang’s research group at Tsinghua University, offers a promising solution for large-scale, two-dimensional grating fabrication. As shown in Figure 2f [94], this design adds an extra mirror to the traditional Lloyd’s mirror setup, facilitating the creation of extensive grating structures. Researchers like Shimizu et al. have extensively studied the mechanisms and applications of two-axis Lloyd’s mirror interferometers, which can be categorized into orthogonal and non-orthogonal configurations based on the mirrors’ relative positions. Figure 3c illustrates a standard orthogonal two-axis Lloyd’s mirror interferometer designed for the high-throughput fabrication of nanoscale two-dimensional gratings with uniformity across large areas [124]. However, additional interference components, caused by reflected beam interactions, can affect pattern uniformity. To mitigate this, polarization directions of the beams are orthogonalized through polarization modulation during exposure. Figure 3d introduces a spatial full-polarization tracking technique, which optimizes exposure conditions by minimizing non-orthogonality between reflected beams while maintaining the necessary interference levels [124]. This approach successfully fabricates two-dimensional grating structures with periodicities of 1 µm, with an exposure apparatus using the orthogonal two-axis Lloyd’s mirror interferometer achieving periodicities of 1076 nm along the X-axis and 1091 nm along the Y-axis.

For broader grating period ranges, Figure 3e presents a two-axis Lloyd’s mirror system with 1/2 wave plate polarization modulation [93,125,126,128]. While this enhances interference control, it also complicates the optical path and reduces the exposure beam’s effective area. In response, Figure 3f introduces a passive polarization holographic lithography system, using a dielectric, film-based polarization modulation to streamline the exposure setup and achieve uniform, large-area (30 mm × 30 mm) array structures with adjustable periods ranging from 742 to 1500 nm [40]. The system’s scalability is further enhanced by integrating multiple dielectric films, providing greater tunability in grating periodicity. Figure 3g illustrates a non-orthogonal, two-axis Lloyd’s mirror interferometer integrated with a polarization modulation control unit [126]. This setup employs a wavefront division technique, splitting the primary laser beam into three distinct beams: a direct beam hitting the substrate, an X-beam reflecting off the X-mirror, and a Y-beam reflecting off the Y-mirror. The X- and Y-mirrors are angled at 90° + *θX* and 90° + *θY* (with *θX*, *θY* > 0°), respectively, while the primary beam’s incidence is perpendicular to the substrate within the interferometer. The superposition of the reflected X- and Y-beams with the direct beam on the substrate surface creates a two-dimensional interference fringe pattern. This non-orthogonal configuration avoids the low spatial frequency stripes typical of orthogonal setups due to the absence of multiple reflections. Additionally, the perpendicular incidence of the primary beam simplifies the alignment of the apparatus. The angles *θX* and *θY* can be adjusted to control the periodicity of the interference fringe patterns in both X and Y directions.

In a non-orthogonal, two-axis Lloyd’s mirror interferometer, large-area, two-dimensional gratings are achieved using oversized X and Y mirrors and an expanded primary laser beam. Figure 3h shows the apparatus for fabricating these gratings, which includes a large-aperture lens to correct and expand the laser beam from a spatial filter assembly (a pinhole and objective lens) [126]. This arrangement allows the interferometer to cover an area of 100 mm × 100 mm. However, the increased beam size reduces the substrate light intensity, necessitating longer exposure times to achieve the required dosage for the photoresist. Despite this, the non-orthogonal configuration’s wavefront division offers resilience against perturbations from the short, non-common optical paths of the subsidiary beams, ensuring consistent grating patterns. A significant challenge of the configuration in Figure 3h is the need for a comprehensive polarization control unit. Producing a large half-wave plate with minimal delay variation is challenging, which can affect polarization modulation. This issue is addressed by incorporating a Galilean beam expander, as shown in Figure 3i. This setup involves a two-step beam expansion process. First, the laser beam is collimated through a spatial filter and then precisely modulated using two half-wave plates with minimal retardance variation. Next, a pair of meniscus lenses and a large aperture collimating lens further expand the modulated beam, generating the primary beam for pattern exposure. Validation confirms that this arrangement reduces pattern distortions and improves the fidelity of the fabricated structures. Another challenge with the non-orthogonal, two-axis Lloyd’s mirror setup is the non-uniform light intensity across the expanded main laser beam. Typically, the laser beam from a spatial filter has a Gaussian profile, which can lead to significant intensity variations after expansion. To address this, a beam shaper with diffractive optical elements (DOEs) is implemented [127,129]. The beam undergoes a three-step expansion process: first, it is collimated through a spatial filter; second, it is shaped into a flat-top intensity distribution using the beam shaper; and third, it is further expanded with meniscus lenses and a large aperture collimating lens. This approach ensures a high-intensity, uniformly distributed laser beam, facilitating the creation of two-dimensional grating patterns with consistent amplitude over large areas. Figure 3j depicts the non-orthogonal, two-axis Lloyd’s mirror interferometer with polarization modulation control and a beam shaper, capable of producing 100 mm × 100 mm gratings in a single exposure process.

### 2.4. Concave Grating Fabrication

Concave gratings are essential optical components widely used in spectral analysis, optical communication, and others. Their ability to both disperse and focus light simplifies optical pathways [127], facilitating the design of compact and lightweight spectroscopic instruments. Traditionally, convex gratings are produced using mechanical ruling, a labor-intensive and time-consuming process. Holographic fabrication methods use interference from two coherent point sources to create grating patterns with variable line spacings. However, fabricating gratings with micrometer-scale line spacings presents challenges. Larger line spacings require the closer placement of point sources [1,9,130,131], which complicates physical spatial filtering and limits the method’s effectiveness [132,133,134,135,136]. To overcome this, Zhou et al., from Tsinghua University, developed a novel dual-beam interference lithography (IL) technique. They improved the conventional dual-beam lithography setup by incorporating a concave lens in front of the optical paths to separate the spatial filters. This configuration maintains the symmetry of the images and ensures high-quality interference patterns. Figure 2g illustrates the operational principle of a concave grating spectrometer (dashed lines) and the optical path for recording the grating pattern (solid lines) [76]. Light entering through the entrance slit, A, is directed onto the concave grating, where it is dispersed, focused, and directed towards a linear detector array, B. The X-axis is aligned with the grating’s normal plane, and the XOY plane, or dispersion plane, is a plane of symmetry. The origin, O, is at the center of the grating in the Cartesian coordinate system. Points C and D denote the two-point sources, while A, B, B1, and B2 are located within the meridional plane XOY. Point P represents an arbitrary location on the grating. As shown in Figure 2g, the grating’s line spacing and the positions of the point sources C and D are defined by specific equations. The line spacing is maximized when the incident angles *θ_C_* and *θ_D_* of the light sources are nearly identical. To achieve larger line spacings, minimizing the angular disparity between *θ_C_* and *θ_D_* is crucial.

(3) $$d=\frac{\lambda }{\left|\sin\theta_{c}-\sin\theta_{D}\right|}$$

Figure 2h depicts the interferometric lithography system used for fabricating concave gratings [96]. A laser beam is divided into two by a polarizing beam splitter, with each beam being shaped and expanded through spatial filters. The polarization directions of the beams are adjusted using half-wave plates to make the interference pattern clearly visible. The positions of the point sources formed by spatial filters 1 and 2, as well as the concave substrate, are precisely adjusted. By using a concave lens to separate the point sources initially, the LIL method effectively overcomes the line spacing limitations of traditional systems. This technique produced a concave grating with a line spacing of approximately 3.8 μm, which was then integrated into a miniaturized spectrometer. The increased line spacing resulted in a 66.5% reduction in detector length while achieving a resolution of better than 1.5 nm over a broad spectral range (360 to 825 nm). This advancement is significant for the development of more compact and higher-resolution spectrometers.

### 2.5. Exposure Field Drift Control

**Figure 4:**
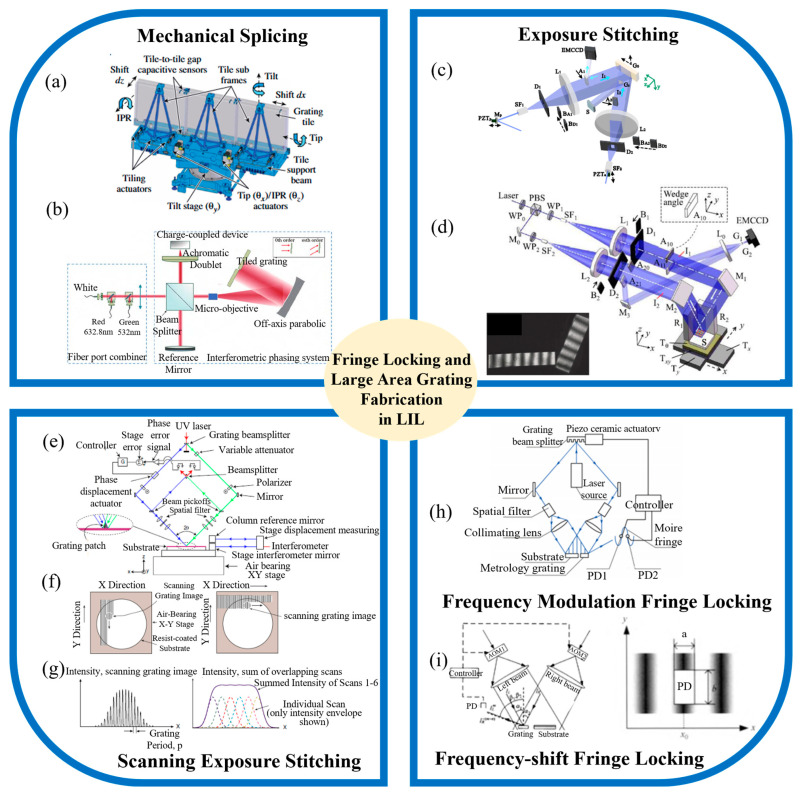
Key techniques in fringe locking for large area grating fabrication: (**a**) large-aperture grating tiling by interferometry for petawatt chirped-pulse amplification systems [137]; (**b**) accurate adjustment technology for longitudinal piston error in echelle grating tiling [139]; (**c**) fringe locking based on latent image gratings for splicing of large-area 1-D gratings [140]; (**d**) fringe locking based on latent image gratings for splicing of large-area 2-D gratings [141]; (**e**) a schematic diagram of a fine-beam scanning exposure system [95]; (**f**) two modes of scanning exposure technology: scanning along raster lines and scanning along raster vectors [142]; (**g**) the intensity distribution of fine beams and the effect of multiple scans [95]; (**h**) the principle of frequency modulation fringe locking; (**i**) the principle of frequency-shift fringe locking [143].

During interference lithography exposure, external disturbances such as temperature fluctuations, humidity changes, and vibrations can cause drift in the exposure interference field. This drift can affect the exposure contrast and impact parameters such as grating groove profiles and duty cycles. To mitigate this drift, either passive isolation methods (e.g., creating stable environmental conditions, enhancing optical platform isolation, and isolating the exposure optical path) or active control solutions can be employed. The fringe-locking technique, a prominent active control method, allows for the real-time phase monitoring of the exposure interference field and uses compensatory elements to keep the phase stable, relative to the substrate. This technique effectively compensates for disturbances that passive methods cannot eliminate, ensuring phase stability during exposure. Its application is particularly significant for maintaining stability in large-scale grating fabrication. To address the limitations imposed by lens apertures in exposure systems, researchers worldwide have proposed stitching together multiple smaller gratings to form a large-diameter composite wavefront. For instance, Figure 4a shows that the research at the University of Rochester used an interference adjustment method to assemble three 0.5 m diameter gratings to replace a 1.5 m diameter grating [137]. By precisely adjusting the position and orientation of the sub-gratings, including their translational and rotational degrees of freedom, they managed to minimize the overall diffraction wavefront distortion. In 2022, Wang et al., from the Nanjing Institute of Astronomical Optics and Technology, developed a method to detect and adjust displacement errors in grating stitching using a Michelson interferometer system combined with white light and two-wavelength measurement techniques, as shown in Figure 4b. Their results, verified through simulations and experiments, demonstrated that the displacement error in the stitched gratings was less than 6 nm, meeting the co-phase detection requirements for large gratings and supporting future advancements in stitching larger diffraction gratings.

Mechanical stitching leverages the lower cost and higher precision of small-diameter gratings to address the challenges of large grating fabrication. However, it requires the repeated use of high-precision control techniques, places high demands on support stability, and incurs spectral losses at the seams between gratings. To address these issues, laser interferometry-based exposure stitching technology has been proposed, which also relies on fringe-locking for precise stitching. Significant advancements have been made internationally in fringe-locking systems for holographic lithography, with commercial solutions now available. Odhner Holographics in the U.S. has introduced the Stabilock II, an integrated fringe-locking device that uses a photodetector to detect phase drift and a piezoelectric mirror system for compensation, achieving a phase-locking accuracy of 0.05λ and a compensation range of ±5 μm. This system is used in holographic grating fabrication. PGL’s nano ruler, based on MIT’s fine beam scanning system, forms a heterodyne interference signal by splitting part of the exposure beam. It monitors phase drift with a photodetector and achieves phase compensation using an acousto-optic modulator and substrate displacement, achieving a phase-locking accuracy of 0.01λ and a long-term repeatability of 1.3 nm over one hour [138]. This high precision and repeatability provide a stable exposure field for large-area grating fabrication.

Zeng et al. at Tsinghua University have proposed a self-referencing fringe-locking method based on latent image grating interference fringes [141,144]. This method uses an EMCCD camera to record the fringes and determines the phase drift by analyzing the light intensity at sampled points. A PZT mounted on a reflective mirror adjusts the phase by altering the optical path difference, as shown in Figure 4c,d [47,145]. This approach avoids external components, minimizing measurement errors and exposure energy loss [96,140]. However, the low diffraction efficiency of latent image gratings necessitates high sensitivity in imaging equipment and stringent control over stray light, which limits real-time control frequency.

The scanning exposure method, which combines holographic exposure and laser direct writing, has recently emerged as a novel technique in holographic lithography. This method utilizes two coherent beams, each with a millimeter-scale diameter, to generate interference fringes for scanning the exposure of a moving substrate. This approach was exemplified by Schatternburg et al. at the Massachusetts Institute of Technology [95,138,146,147,148,149,150]. The fine-beam scanning exposure system, illustrated in Figure 4e [95], involves splitting part of the exposure beam and employing an acousto-optic modulator to produce a heterodyne signal for measuring and adjusting the phase difference between the two beams. A plane mirror is positioned on the translation stage, and its position is measured using a red-light interferometer. These adjustments are made using precise electronic components. The scanning exposure technique has two modes: scanning along the grating lines and scanning along the grating vector, as shown in Figure 4f [142]. To achieve a uniform scanning exposure, due to the Gaussian intensity distribution of the fine beam, multiple scans are necessary, as depicted in Figure 4g [151]. This method has been used to fabricate a scanning exposure grating measuring 910 mm × 420 mm, with a period of 1/1740 mm and diffraction wavefront peak-to-valley values better than λ/3 [152]. Additionally, researchers from the Changchun Institute of Optics, Fine Mechanics, and Physics have developed a moiré-based fringe-locking system, illustrated in Figure 4h [153]. This system uses moiré fringes generated by a measurement grating within the exposure light field for phase monitoring. A beam-splitting grating divides the light, and any drift in the exposure interference field is corrected by actuating the beam-splitting grating with a piezoelectric ceramic element. The system utilizes the Doppler effect caused by the grating’s movement to adjust the phase of the exposure light field, achieving compensation. The experimental results show a locking precision of better than 0.021 interference fringe cycles. Compared to the piezoelectric ceramic mirror method, this system has a reduced impact on period regulation but introduces external components, which lowers the laser power utilization.

Researchers at the Changchun Institute of Optics, Fine Mechanics and Physics, including Song et al., have also proposed a frequency-shifting fringe-locking system, shown in Figure 4i [154]. This system fixes a measurement grating within the exposure light field to create reference moiré fringes. Photodiodes within these fringes monitor optical power in real-time to detect phase drift. Phase drift is corrected by adjusting the carrier frequency of acousto-optic modulators (AOMs) in both beams, leveraging the frequency-shifting effect. This system offers a broad adjustment range and rapid compensation, achieving phase correction of less than 0.02 cycles. However, the use of AOMs limits the system to the first order of diffracted light for exposure, leading to significant power loss. Moreover, this drift monitoring method is sensitive to fluctuations in laser power and ambient light changes. It also faces challenges in accurately adjusting for substrate attitude errors, as relying solely on one-dimensional brightness information is insufficient. In recent years, several teams from institutions such as Tsinghua University, Suzhou University, and the Changchun Institute of Optics, Fine Mechanics and Physics, Chinese Academy of Sciences, have also proposed various fringe-locking system solutions, improving the fringe-locking accuracy by refining phase monitoring light paths and compensation element designs. With the development of computer technology and increased computational power, image processing algorithms and digital controller algorithms have also been applied in fringe-locking systems, further enhancing the controllability of fringe-locking systems. These studies have created possibilities for the application of fringe-locking technology in the field of holographic lithography, with significant potential for application and promotion.

## 3. Key Developments in Grating Interferometry

**Figure 5:**
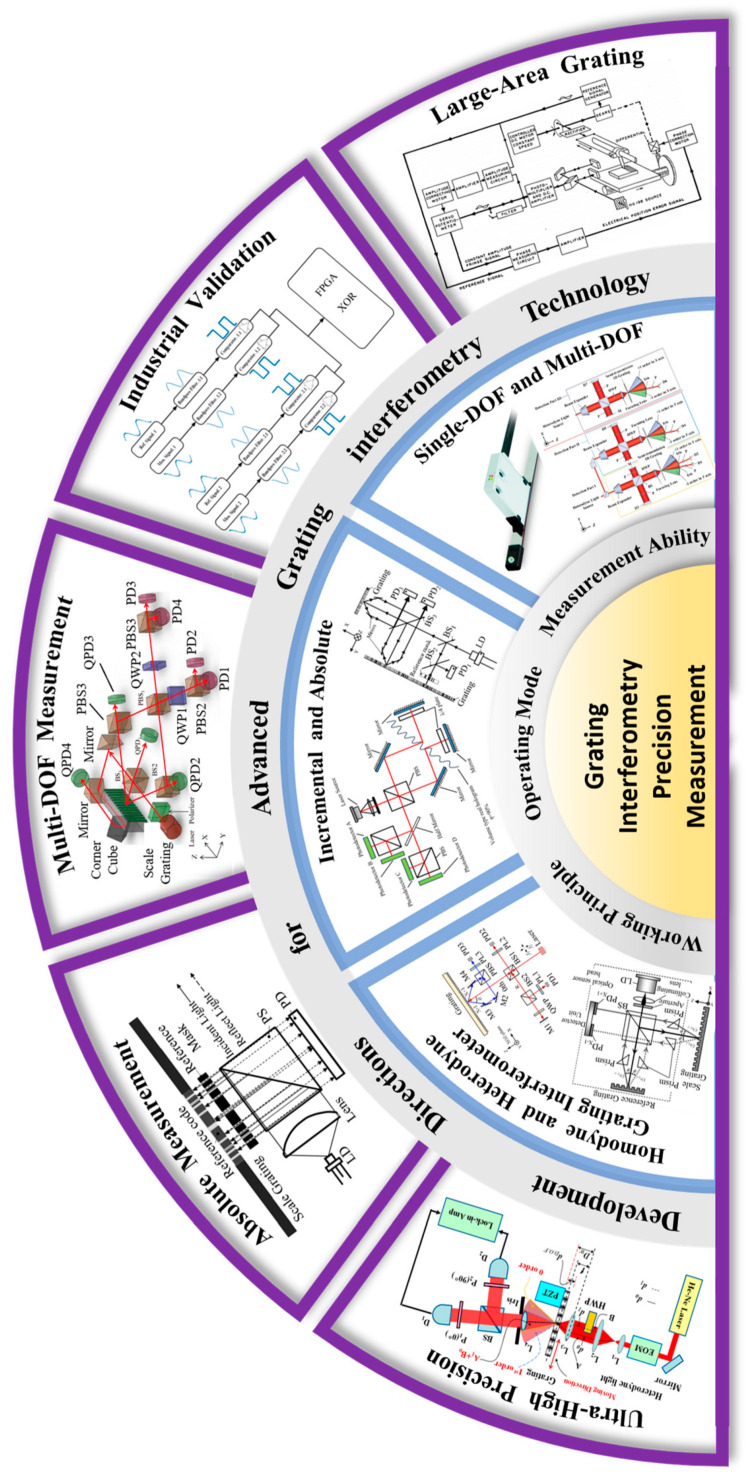
Key developments in grating interferometry [166,167,168,169,170,171,172,173,174,175,176,177].

Since Teimel introduced grating interferometry technology into the field of industrial measurement [155], it has garnered significant attention from researchers worldwide [156,157,158,159]. Typically, grating interferometry is classified based on measurement principles or degrees of freedom (DOF), a categorization approach that facilitates a comprehensive understanding of the technology’s overall development. However, in recent years, with the continuous development of grating interferometry technology, it has increasingly advanced towards directions such as higher precision [32,156], absolute measurement [160,161], multi-degree-of-freedom (multi-DOF) measurement [162,163], and industrialization [164,165]. As shown in Figure 5, these advancements are crucial for achieving higher accuracy and broader application ranges.

Taking the development direction of grating interferometry technology as the starting point, this paper classifies and discusses the structural characteristics, working principles and development prospects of this technology in different directions, which is of great significance for the subsequent development of this research field. Such an approach not only provides a clearer understanding of the current state of the technology but also highlights key areas for future innovation and application, thereby driving further advancements in grating interferometry.

### 3.1. High-Precision Measurement

High-precision measurement has consistently been a mainstream development direction in the field of grating interferometry [178,179,180]. This focus has become even more pronounced, with continuous technological advancements. In the semiconductor processing industry, for instance, manufacturing precision is now advancing towards near-atomic scales, which places increasingly stringent demands on measurement accuracy. Currently, the primary methods for achieving high-precision measurements include multiple optical subdivision [181,182], multiple electronic subdivision [183,184], the use of gratings with smaller periods [178,185,186], and laser frequency stabilization [166,187,188]. A detailed introduction to these methods will be provided below.

#### 3.1.1. Multiple Optical Subdivision

From the perspective of measurement principles, the interference measurement of unidirectional displacement can be achieved by using a reference grating and a measurement grating [189,190,191,192], each corresponding to a specific diffracted beam. Some researchers have utilized additional diffraction orders for synchronous measurement, employing multiple optical subdivisions of the phase signals generated by interference to enhance measurement accuracy [193,194]. 

**Figure 6:**
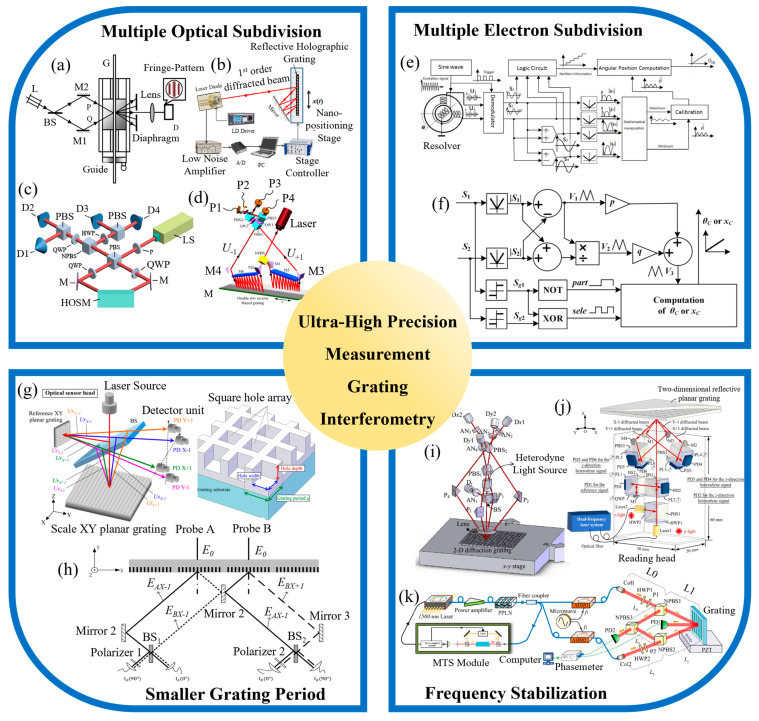
Ultra-high precision measurement grating interferometry: (**a**) a schematic diagram of the grating interferometer with 10-fold optical subdivision [181]; (**b**) a schematic diagram of the improved laser self-mixing grating interferometer (SMGI) system [195]; (**c**) a schematic diagram of the grating interferometer with 8-fold optical subdivision, based on special prism structure [196]; (**d**) a schematic diagram of the grating interferometer with 18-fold optical subdivision using dual-row counter-blazed grating configuration [197]; (**e**) a schematic diagram of the new resolver-to-digital conversion method [203]; (**f**) a schematic diagram of the electronic interpolation interface, based on linear subdivision method [205]; (**g**) a schematic diagram of the three-axis surface encoder with short period planar grating and the schematic of the grating structures for the XY planar grating [89]; (**h**) a schematic diagram of the linear encoder with a dual-probe reading head [206]; (**i**) a 2-D in-plane displacement measurement system, based on heterodyne grating interferometry [207]; (**j**) a schematic diagram of the heterodyne, 3-DOF grating interferometer [172]; (**k**) a schematic diagram of the symmetric oblique incidence heterodyne grating interferometer [208].

In 2008, Chu et al. proposed a long-range grating interferometer system [181], as shown in Figure 6a. By adjusting the incident angle of the beams, the ±5th-order diffracted beams interfered to form a fringe pattern, achieving a 10-fold optical subdivision. The system ultimately reached a resolution of 1 nm, with a measurement accuracy of better than 10 nm. However, this method suffers from weak diffracted fringe energy, and the measurement accuracy is highly dependent on the quality of the fringe signal and the precision of the fringe subdivision algorithm. In 2017, Xing et al. proposed a spatially separated heterodyne grating interferometer [182], utilizing a quadruple optical subdivision design. This innovation successfully reduced the periodic nonlinear error to less than 0.086 nm. Later, Zhou et al. also introduced a heterodyne interferometric measurement system with quadruple optical subdivision [32], achieving a resolution of 2 nm within a linear motion range of 10 mm and a measurement error of less than 15 nm. In the same year, Guo et al. proposed an improved laser self-mixing grating interferometer (SMGI) system [195], as shown in Figure 6b. This system enhanced the measurement resolution by employing multiple diffractions and achieved higher optical subdivision through repeated reflections and diffractions between a mirror and the grating. The system’s resolution was better than 5 nm, and with six diffraction events, a six-fold optical subdivision was achieved. During the micro-displacement measurement experiments, the system demonstrated a measurement accuracy of less than 1.6 nm. 

In 2018, Deng et al. designed a high-order optical subdivision module (HOSM) and a high-line-density grating of 1780 lines/mm using a special symmetric prism structure [196], as shown in Figure 6c. This setup allowed eight reflections and diffractions of the beam on the grating, increasing the number of interference fringes and achieving an eight-fold optical subdivision, quadrupling the subdivision count compared to traditional grating interferometers. Theoretically, this method could achieve a picometer-level resolution, with the experimental results showing a resolution of better than 1 nm. However, this method has high structural requirements for the symmetric prism, and the non-uniformity of the high-line-density grating can introduce significant measurement errors. More recently, in 2023, Zhang et al. proposed a novel design featuring a grating interferometer with a dual-row counter-blazed grating configuration [197], as shown in Figure 6d. By increasing the number of diffractions on the grating surface, they achieved a 16-fold optical subdivision. Within a measuring range of 1.7 mm, the system attained a measurement accuracy of ±90 nm. However, this design has limitations, such as the presence of unusable beams and a reduction in the system’s optical power conversion efficiency to 50% of its original value. Additionally, alignment errors between the grating and mirror, as well as manufacturing errors in the grating, can lead to measurement inaccuracies.

#### 3.1.2. Multiple Electronic Subdivision

During the measurement process, the interference signals being processed are essentially sinusoidal and cosine signals [198]. In addition to optical subdivision, more refined electronic subdivision can be applied in signal processing to enhance measurement accuracy [199,200].

In 2005, Benammar et al. proposed a high-precision resolver-to-DC converter that calculates the absolute value difference between demodulated sine and cosine signals and performs linearization processing, achieving improved linearity and accuracy [201]. In the same year, Tan et al. introduced an adaptive online correction and interpolation method based on a radial basis function (RBF) neural network [199]. This approach uses a two-layer RBF neural network structure: the first stage performs the real-time correction of encoder signals, while the second stage generates higher-order sinusoidal signals, achieving an electronic subdivision level of 4096 times and enabling higher precision in position measurement. However, this method relies heavily on the adjustment and the training of the RBF neural network parameters, and signal noise and imperfect input data can affect the model’s generalization ability and accuracy. 

In 2009, Hu et al. proposed a novel electronic subdivision method that achieved a 16-fold subdivision of quadrature interference signals and direction recognition by constructing two sets of reference signals and utilizing zero-crossing detection, reaching a nanometer-level measurement accuracy [198]. Later, Hoang et al. introduced an improved signal processing method for magnetic encoders, employing an advanced adaptive digital phase-locked loop (AADPLL) and a new pulse interpolator technology [200], which enabled high-precision signal correction and high-resolution quadrature pulse generation, also achieving 16-fold subdivision. In 2014, Ye et al. proposed a precise and robust linearization converter that utilized ratio technology and a dedicated compensation method to convert sine and cosine signals into nearly linear output signals [202], thus achieving high-precision displacement measurement. The experimental results showed that, within a measurement range of 80 mm, the positioning accuracy of the system reached ±0.2 μm, with a nonlinearity error of less than 0.0029 μm. The same year, for angular measurement, Wang et al. introduced a new resolver-to-digital conversion method [203]. The specific structure, as shown in Figure 6e, generated auxiliary sine signals using addition and subtraction operations, resulting in pseudo-linear signal generation and improved angular measurement accuracy. The experimental results indicated that the angular measurement error within a 360-degree range was less than 0.00235 degrees, making it suitable for most high-precision application scenarios. In 2017, Peng et al. significantly enhanced measurement accuracy by employing a multi-layer structure and adjusting the setup to reduce nonlinear responses and cross-interference [204]. Later, in 2019, Zhao et al. proposed an electronic interpolation interface based on a linear subdivision method [205], as shown in Figure 6f. By generating pseudo-linearized signals and constructing compensation signals, they achieved up to 40,000-fold electronic subdivision through electronic interpolation techniques. Within a periodic signal of 80 μm, the theoretical interpolation error was less than 0.018 μm.

#### 3.1.3. Using Gratings with Smaller Periods

When a grating moves by a displacement equal to one grating period, the phase difference of the interference signal changes by 360° [209,210]. Thus, using gratings with smaller periods can effectively increase the level of signal subdivision [211], thereby enhancing measurement accuracy [212,213]. In 2012, Gao et al. designed and manufactured an XY plane grating with a period of 1 μm and proposed a three-axis surface encoder with a short-period plane grating [89], as shown in Figure 6g. The experimental results showed interpolation errors of ±10 nm, ±10 nm, and ±3 nm in the X, Y, and Z directions, respectively, achieving a resolution of better than 1 nm on all three axes. In the same year, using a grating with a period of 570 nm, a three-axis surface grating encoder was proposed, achieving a nanometer-level resolution. In 2016, Lu et al. utilized a short-period grating with a period of 561.8 nm to measure both horizontal and vertical displacements simultaneously, improving measurement accuracy and resolution [214]. The experimental results indicated that, within an 8 mm measurement range, the maximum measurement discrepancies between the grating encoder and the laser interferometer in the horizontal and vertical directions were 60 nm and 80 nm, respectively, primarily due to Abbe errors. Later, in 2019, Li et al. proposed a linear encoder using a 607 nm short-period grating with a dual-probe reading head and spliced short gratings [206], as shown in Figure 6h. The experimental results demonstrated that, within a 20 mm measurement range, the measurement error of the encoder was within ±1 μm, validating the feasibility and high-precision performance of the spliced grating design.

While small-period gratings can enhance measurement accuracy, they require high manufacturing precision [215]. Additionally, for diffraction to occur during measurement, the wavelength of the incident laser must be smaller than the grating period [216,217]. Therefore, smaller grating periods correspond to shorter laser source wavelengths. On the other hand, the smaller the grating period, the larger the diffraction angle, which can hinder the integration of the entire measurement system [218,219].

#### 3.1.4. Laser Frequency Stabilization

In a conventional single-frequency grating interferometer, the measurement signal typically exhibits a DC offset, which can drift due to shifts in the light source. To address this issue, some researchers have conducted in-depth studies on heterodyne light sources [220], using heterodyne frequency stabilization to eliminate the DC offset [188]. In heterodyne systems, the light source emits two laser beams with closely spaced but orthogonal frequencies. The interference of these two beams produces a beat frequency that cancels out the DC offset, thereby enhancing the overall stability of the system, increasing its resistance to interference, and ultimately improving measurement accuracy [187].

In 2008, Hsu et al. proposed a two-dimensional (2-D), in-plane displacement measurement method based on heterodyne grating interferometry [207], as shown in Figure 6i. This method achieved sensitivity at the sub-picometer level and, through a controlled isolation system, obtained a resolution of approximately 0.5 nm over a displacement range of 250 μm. In 2010, Hsieh et al. introduced a novel heterodyne grating interferometer based on a quasi-common-path design [166], achieving a resolution of better than 3 nm and stability within 14 nm over an hour. Later, in 2022, Zhu et al. developed a heterodyne, 3-DOF grating interferometer using a custom-built, 780 nm, dual-frequency laser source [172], as shown in Figure 6j. The experimental results showed that the three-axis resolution was better than 0.5 nm, the short-term repeatability was better than 0.6 nm, the linearity was better than 2 × 10^−5^, and the 300 s stability was better than 5 nm. In the same year, the same team proposed a polarization adjustment module for heterodyne grating interferometers [221], which partially resolved the frequency aliasing problem in heterodyne grating interferometers, further improving measurement accuracy. Subsequently, in 2024, Wang et al. adopted a symmetric oblique incidence structure [208], as shown in Figure 6k, reducing the heterodyne phase errors caused by optical path differences in the interferometer. The heterodyne light source achieved a frequency stability better than 2.6 × 10^−9^ over 5 h, with a resolution of better than 0.3 nm, a repeatability of better than 0.4 nm, and a periodic nonlinearity error of better than 0.3 nm. 

Overall, the measurement accuracy of the grating interferometer can be improved using the four aforementioned methods, but each comes with its own limitations. Increasing the optical subdivision factor may involve the use of other diffraction orders or multiple reflections of a single diffracted beam, both of which can reduce laser energy and decrease the signal-to-noise ratio. Increasing the electronic subdivision factor imposes higher demands on signal processing algorithms and hardware. Using gratings with smaller periods will result in larger diffraction angles, making system integration more difficult, and small-period gratings present challenges in terms of fabrication techniques and processes. Additionally, employing dual-frequency light sources increases system complexity, makes signal resolution algorithms more intricate, and significantly raises costs.

### 3.2. Absolute Measurement

Since the phase signal measured by a grating interferometer exhibits periodic changes with the movement of the grating, most grating interferometers primarily use incremental measurement methods [178,222]. However, there is a growing demand for absolute grating interferometers that can record the absolute position of the grating in real-time. To meet this need, some researchers have employed absolute encoding techniques to develop absolute grating interferometers [223,224,225].

**Figure 7:**
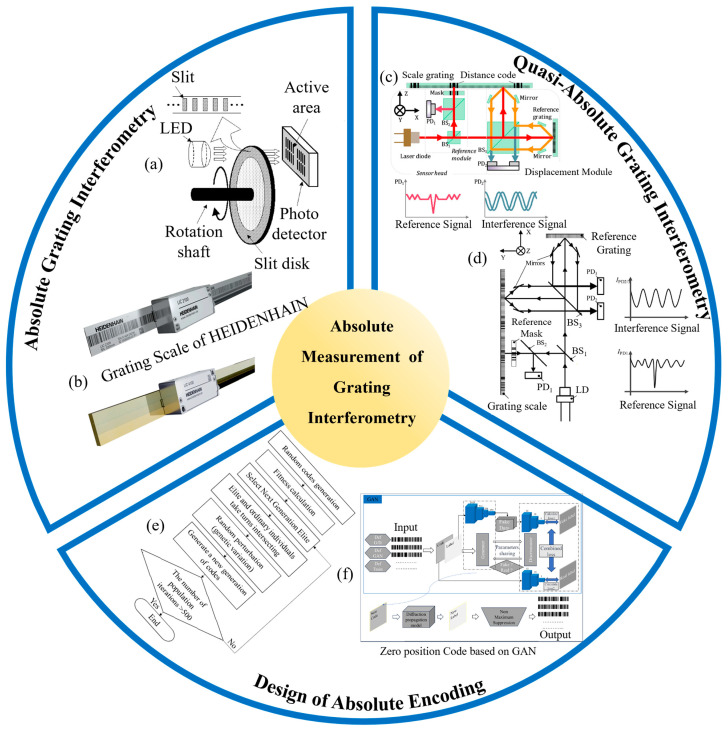
Absolute measurement of grating interferometry: (**a**) a schematic diagram of the grating measurement system, combining multi-track and M-code encoding [226]; (**b**) the absolute linear encoders of HEIDENHAIN [229]; (**c**) a schematic diagram of the absolute optical encoder, based on the hybrid positioning method [228]; (**d**) a schematic diagram of the improved dual-probe absolute linear encoder [174]; (**e**) the binary encoding design method based on the genetic algorithm [230]; (**f**) the zero-position encoding design method based on generative adversarial networks (GANs) [231].

In 2003, Matsuzoe et al. proposed a novel grating measurement method by combining multi-track and M-code encoding [226], as shown in Figure 7a, achieving absolute measurement by integrating interpolation data from two different periods. Some researchers have also employed binary encoding to achieve absolute measurement, which is currently mainly used in quasi-absolute encoding designs and which will be discussed in detail later. Compared to binary encoding, gray code offers higher stability and has advantages in hardware implementation. A notable example is HEIDENHAIN, as shown in Figure 7b, which has developed numerous grating interferometers based on gray code. However, this type of absolute encoding requires encoding the entire range of the grating, resulting in a relatively wide absolute code width, which can lead to lower overall measurement precision.

Some researchers have opted to encode specific regions of the grating more precisely, marking certain positions on the measurement grating to achieve quasi-absolute measurement through position pulse coordinates and incremental displacement. In 2016, Li et al. proposed a dual-probe optical encoder based on an improved single-track grating. The improved grating physically superimposes multiple reference codes on the incremental grooves without requiring additional tracks [167]. The experimental results showed that the positioning accuracy of the reference signal reached 0.5 μm, with a measurement linearity of less than 0.06%. In 2018, Wang et al. introduced an enhanced absolute positioning method using reference code tracks with different pitches, superimposed on the incremental grooves [227]. By combining zero-pulse signals with incremental displacement signals, absolute measurement was achieved. The experimental results demonstrated that the half-peak width of the zero-pulse signal was 29 μm, corresponding to a positioning accuracy of approximately 0.5 μm, with an incremental signal period of 1 μm. In 2019, Shi et al. proposed a new hybrid positioning method for absolute optical encoders by combining reference pulse signals with incremental displacement signals [228], as shown in Figure 7c. Two probes simultaneously read the distance code and the grating grooves; one read head uses a mask that matches the grating code to approximate the marked position with a high accuracy of 0.5 μm, while the other read head utilizes the grating measurement method for nanometer-resolution displacement measurement. Test results indicated that within a range of several tens of millimeters, the repeatability of the positioning accuracy was 10 nm. Building on this, in 2020, Shi et al. further developed an improved linear encoder that employs a dual-probe system to simultaneously measure both incremental and reference signals [174], as shown in Figure 7d. The reference marks are detected using a window fitting method to pinpoint the pulse signal’s peak. The incremental displacement resolution was 15 nm, and the absolute positioning accuracy was comparable to the resolution when the incremental signals were used cooperatively.

As the number of bits in absolute encoding increases, traditional exhaustive methods for finding the optimal encoding are gradually being abandoned. Designing suitable and effective absolute encodings has become a research focus for some scholars [232]. In 2023, Wang et al. proposed a code-coupling method for optimizing absolute encoding [233]. By optimizing various parameters, such as the distance and angle between the mask and the grating and the width of the encoding units, they developed a multi-parameter model to simulate and analyze the optimal operating parameters. The encoder’s positioning accuracy under different parameter settings could reach micron or sub-micron levels. In the same year, he introduced a binary encoding design method based on genetic algorithms [230], as shown in Figure 7e, which could quickly generate suitable encodings to meet high-resolution measurement needs. The fitness of the encoding was significantly improved, ultimately achieving a nanometer-level absolute positioning accuracy. Following this, in 2024, Wang et al. used deep learning-based generative adversarial networks (GANs) to optimize zero-position encoding design [231], as shown in Figure 7f, generating a high-quality, 150-bit, binary, zero-position encoding. Compared to traditional methods, this method improved the positioning accuracy by approximately 129%, providing an effective design method for binary absolute encoding.

Although absolute encoding can facilitate absolute measurements relatively easily, there is still no optimal design method, particularly for high-bit absolute encoding. When conducting multi-degree-of-freedom measurements, changes in the relative position between the absolute encoder and the measurement grating can introduce significant measurement errors.

### 3.3. Multi-Degree-of-Freedom Measurement

Compared to laser interferometers, grating interferometers use the grating pitch as a reference, offering higher stability and stronger resistance to interference [172]. Additionally, due to their symmetrical structure, grating interferometers can achieve multi-DOF measurements without needing multi-DOF combinations [162,234,235], effectively avoiding Abbe errors. This gives them a significant advantage in the field of multi-DOF measurement, making it a predominant direction in current development [177,236].

**Figure 8:**
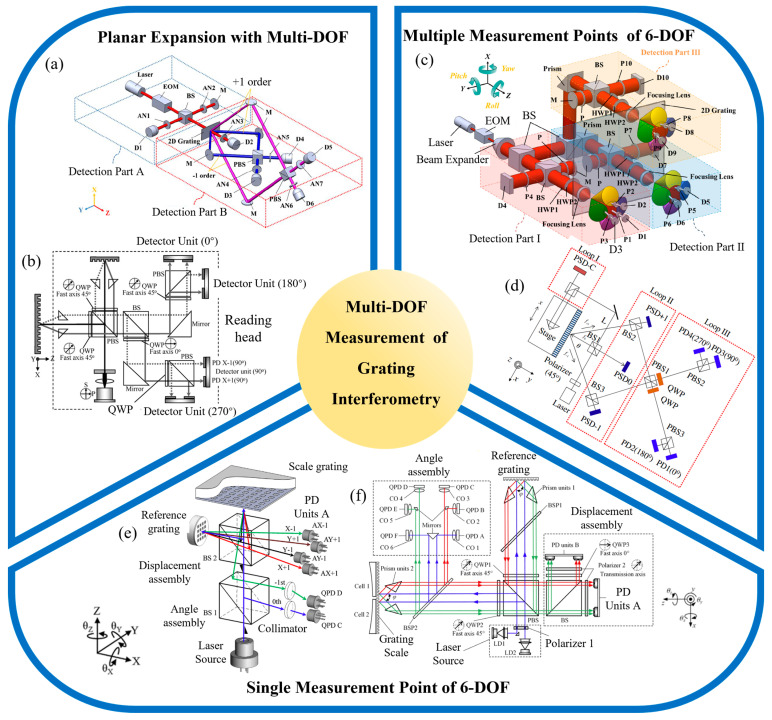
Multi-DOF measurement of grating interferometry: (**a**) a schematic diagram of the heterodyne 3-DOF grating interferometer [239]; (**b**) a schematic diagram of the 2-D linear encoder, based on two-dimensional grating [237]; (**c**) a schematic diagram of the three measurement points of the 6-DOF grating interferometer [241]; (**d**) a schematic diagram of the two measurement points of the 6-DOF grating interferometer [242]; (**e**) a schematic diagram of the one measurement point of the 6-DOF grating interferometer [243]; (**f**) a schematic diagram of the dual-channel, 6-DOF grating interferometer [244].

A typical extension involves planar multi-DOF systems. In 2010, Gao et al. proposed a 2-DOF linear encoder based on a 2-D grating [237], as shown in Figure 8b, which can simultaneously measure the X-axis position of a precision platform and the Z-axis straightness perpendicular to the motion axis. This system achieves biaxial displacement measurement by superimposing interference signals from positive and negative first-order diffracted beams. Experimental data indicate that the encoder achieves a resolution of better than 1 nm in both the X and Z axes, providing sub-nanometer measurement precision. Subsequently, in 2011, the same research group introduced a two-DOF linear encoder utilizing a mosaic grating to simultaneously measure the X-axis position and Z-axis straightness [238]. This encoder achieved a resolution of 5 nm in the X direction and 1 nm in the Z direction, with a peak-to-valley nonlinearity error within ±50 nm in the X direction. In 2013, Hsieh et al. proposed a heterodyne, three-DOF grating interferometer, employing photoelectric modulation to generate beat-frequency beams [239], as shown in Figure 8a. This system achieved a nanometer-level resolution in three-dimensional micro-displacement measurements, with horizontal and vertical straightness errors of approximately 350 nm and 400 nm, respectively. The stability of the in-plane detection configuration was better than 30 nm, while the out-of-plane detection configuration exhibited a stability of around 40 nm. In 2021, Zhu et al. presented a 2-DOF grating interferometer that employs a dual-phase optical path configuration and a simplified dual-phase differential decoding algorithm [240]. This grating encoder is capable of displacement measurement in both the X and Z directions. The experimental results demonstrated that, within a measurement range of 4 mm, the grating encoder’s measurement error remained within ±1 μm. The maximum measurement error within a single period was 2 nm, and the system resolution could reach 50 pm.

Some researchers have extended displacement measurement techniques to measure multiple points on a target object, utilizing redundant information to achieve the spatial 6-DOF pose measurement of the object. In 2000, Fan et al. employed four Laser Doppler sensors and two quadrant photodetectors to detect six motion errors of an X-Y stage in real-time [245]. This measurement system achieved a linear positioning accuracy of better than 0.1 mm within a measurement range of 200 mm. In 2011, Lee et al. proposed a two-point, 6-DOF grating interferometer [242], as shown in Figure 8d, which integrates diffractive optical elements; corner reflectors; two-dimensional, position-sensitive detectors; and auxiliary optical components to simultaneously measure translational and rotational errors. The experimental results showed that the encoder achieved resolutions of better than 0.4 nm (translation) and 0.03 arcseconds (rotation). Later, the same research group introduced another two-point grating interferometer for 6-DOF error measurement [168], achieving displacement resolutions of better than 0.4 nm in the X direction and of better than 20 nm in the Y and Z directions, with a rotational error of less than 0.03 arcseconds. In 2015, Hsieh et al. proposed a three-point, heterodyne, 6-DOF grating interferometer [241], as shown in Figure 8c, which utilizes multi-point redundant measurements to achieve spatial 6-DOF measurement, with displacement and angular resolutions reaching 2 nm and 0.02 arcseconds, respectively. However, the overall optical path structure is relatively complex, making integration and miniaturization challenging.

Some researchers have utilized the characteristics of grating diffraction to achieve single-point, 6-DOF measurements [246]. In 2013, Li et al. was the first to combine XYZ displacement measurement with angular measurement by using a beam splitter to separate the two modules [243], as shown in Figure 8e. They introduced a single-point, 6-DOF grating interferometer, achieving a displacement resolution of up to 1 nm and an angular resolution of better than 0.3 arcseconds. In 2019, Gao et al. conducted an in-depth study of crosstalk errors in this structure and provided a detailed discussion of the compensation methods [247]. Subsequently, in 2021, building on this foundation, Yu et al. proposed a dual-channel, 6-DOF grating interferometer [244], as shown in Figure 8f. This system utilizes two sets of gratings and light sources to measure the relative pose of two adjacent gratings, achieving an angular resolution of better than 0.02 arcseconds and a displacement resolution of better than 50 nm. The displacement measurement error of the dual reading head configuration is less than ±0.1 μm, and the angular measurement error is less than ±0.1 arcseconds. Although grating interferometers have certain advantages in achieving multi-DOF measurements, the overall structure of grating encoders is relatively complex. Additionally, the measurement accuracy of 6-DOF grating interferometers has been constrained by crosstalk between the different degrees of freedom [248]. Optimizing the structure of multi-DOF grating interferometers and reducing coupling errors are key areas for future research [249].

Although grating interferometers offer significant advantages in achieving multi-DOF measurements, when a single grating interferometer is used to measure the six degrees of freedom of a spatial object, the coupling error model between different degrees of freedom becomes highly complex. Achieving a high-precision displacement resolution remains a major challenge that needs to be addressed.

### 3.4. Industrialization Modules of Grating Interferometry

Grating interferometers have significant advantages in industrial applications, including high precision, non-contact measurement, and real-time monitoring capabilities, making them well-suited for high-precision displacement monitoring in dynamic and harsh environments. Some researchers have conducted studies on the industrialization of this technology, focusing on the development of diffraction light modulation modules [250], integrated interferometric optical path modules [251], and the design and integration of signal processing systems [252].

#### 3.4.1. Mature Industrial Products

**Figure 9:**
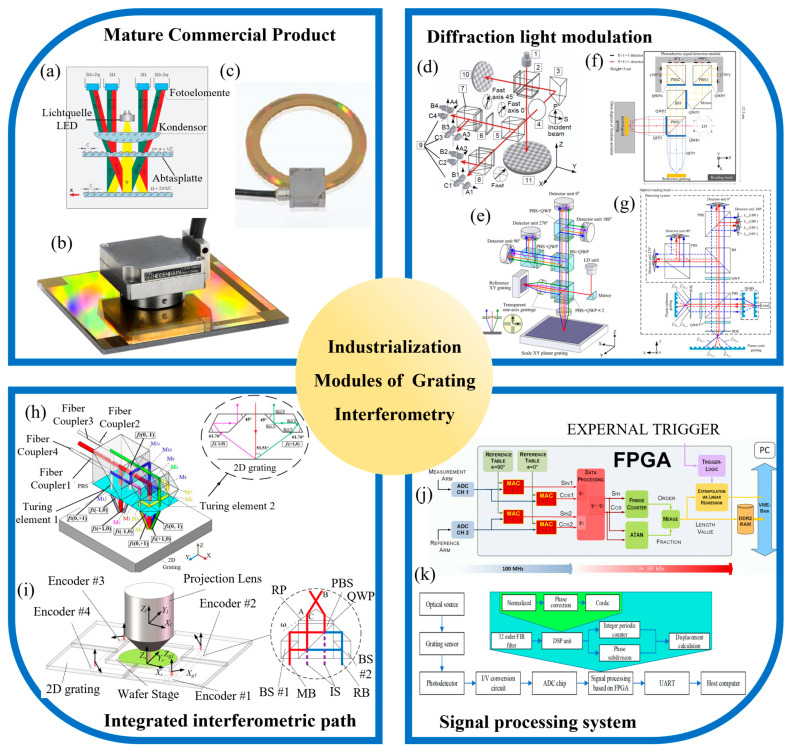
Industrialization modules of grating interferometry: (**a**) a schematic diagram of the LF185 one-dimensional grating scale of HEIDENHAIN [254]; (**b**) a schematic diagram of the PP281 two-dimensional grating scale of HEIDENHAIN [256]; (**c**) a schematic diagram of the BS78 one-dimensional grating scale of Magnescale [258]; (**d**) a three-DOF grating interferometer, based on lens module collimation [259]; (**e**) a three-DOF grating interferometer, based on transmission grating collimation [89]; (**f**) a three-DOF grating interferometer, based on roof prism [260]; (**g**) a three-DOF grating interferometer, based on the Littrow configuration [165]; (**h**) a 2-D grating displacement measurement system, based on dual spatial heterodyne optical path interleaving [251]; (**i**) a four-grating interferometer system of the wafer stage and the concept of the encoder [261]; (**j**) the system structure of the laser interferometer signal processing platform at the PTB laboratory [262]; (**k**) a next-generation grating interferometer data processing system, based on the FPGA platform [164].Magnescale [263], a leading company in ultra-precision measurement technology in Japan, uses semiconductor laser beams to generate interference fringe variations through gratings for displacement measurement. Its products feature the advantages of non-contact measurement and strong anti-interference capabilities, making them widely used in precision machining and similar applications. A representative product is the BS78 linear scale model [258], as shown in Figure 9c, which offers a maximum motion speed of 400 mm/s and a resolution of 17 picometers. Within a measurement range of 40 mm, it achieves a measurement accuracy of better than ±40 nm.

In the field of grating interferometry technology, several high-precision mature products have been developed, primarily by companies like HEIDENHAIN and Magnescale. HEIDENHAIN [253], in particular, has introduced two types (enclosed and open types) of linear grating interferometers to meet the measurement needs in CNC machine tools and semiconductor production. The enclosed linear scales, such as the LC, LF, LB, and LS series, support both incremental and absolute measurements and feature a compact design. For example, the LF185 series achieves a measurement accuracy of ±3 μm within a 440 mm travel range [254], as shown in Figure 9a. The open linear scales offer higher precision, primarily targeting semiconductor metrology applications. For instance, the LIP382 model achieves an accuracy of ±0.5 μm over a 150 mm travel range [255], with a measurement step size of 1 nm. Additionally, HEIDENHAIN has developed two-axis incremental scales like the PP281 [256], as shown in Figure 9b, designed for in-plane, 2-DOF measurements with an accuracy of ±2 μm over a measurement range of 68 mm × 68 mm. Apart from linear scales, HEIDENHAIN has also introduced high-precision angular encoders based on gratings, such as the ECA 4402 model [257], which offers a measurement accuracy of ±3 arcseconds, with an outer diameter of 104.63 mm and an inner diameter of 70 mm. These products exemplify the advanced capabilities and broad applications of grating interferometry technology in various high-precision industrial fields.

#### 3.4.2. Diffraction Light Modulation Module

The primary function of the diffraction light modulation module is to modulate the diffracted light from both the reference grating and the measurement grating, ensuring that the diffracted beams from different gratings can return to the interferometric optical path. Researchers have proposed various modular structures for the modulation of diffracted light. The commonly used diffraction light modulation methods include lens modules [264], multi-prism module modulation [244], roof prism modulation [260], mirror module modulation [265], Littrow configuration modulation modules [266], and transmission grating modulation [89]. These methods are designed to optimize the alignment and quality of the interference pattern, thereby enhancing the measurement accuracy and reliability of grating interferometry systems in various applications.

Using the lens module for collimation requires placing the reference grating and the measurement grating at the focal points of the lens. By leveraging the lens’s properties to collimate the diffracted beams, this approach enables a structural design with a long working distance. In 2007, Gao et al. utilized a lens module to collimate the diffracted light and [259], for the first time, replaced the traditional Michelson interferometer mirrors with a triaxial displacement sensor. They used two sinusoidal XY grid mirrors with the same pitch and amplitude to generate interference signals for displacement measurements along the X, Y, and Z axes. The experimental results confirmed that this method achieved nanometer resolution on all three axes.

The multi-prism module, composed of two or four identical prisms, is used to collimate the ±1st-order diffracted beams from the grating through a single total internal reflection and two refractions, which are then used for interferometric measurement. This diffraction light modulation method is the most widely used in grating measurement technology. In 2010, Gao et al. proposed a 2-DOF, zero-difference grating interferometer reading head. This design utilized a prism group to collimate the grating’s diffracted light [264], and the interference superposition of the positive and negative first-order diffracted beams from the reference and scale gratings was used to resolve the displacement signals. The final optical reading head measured 50 mm (X) × 50 mm (Y) × 30 mm (Z), and testing demonstrated that it could achieve sub-nanometer resolution along both the X-axis and Z-axis. Many other designs also utilize multi-prism collimation, and they have been discussed earlier [237,243,244], so they will not be repeated here.

The multi-prism module can individually modulate four diffracted beams, and building on this, some researchers have opted to use a roof prism to modulate all four beams simultaneously [267]. This approach results in a more compact structure, facilitating miniaturization. However, the parameters of this system need to be customized according to optical system specifications, such as grating period, working distance, and laser source wavelength, requiring high precision in the manufacturing process. In 2023, Wang et al. proposed a compact [260], high-precision, three-degree-of-freedom grating encoder based on a roof prism, with an overall reading head size of 12.3 cm (X) × 3 cm (Y) × 7.7 cm (Z), and further miniaturization potential. The test results demonstrated the capability to simultaneously measure three degrees of freedom within the ranges of X-250 μm, Y-200 μm, and Z-100 μm, with an average primary displacement measurement accuracy of below 500 nm, and minimum and maximum errors of 0.0708% and 2.8422%, respectively.

The multi-mirror module consists of two or four identical mirrors. Unlike the prism module, it achieves the collimation and the modulation of the grating’s diffracted light with just a single reflection, making the overall structure and parameter selection simpler and easier to adjust. However, reflections can sometimes result in a half-wavelength loss, which needs to be carefully considered during the initial system design. In 2014, Zhu et al. proposed a new heterodyne grating interferometer system using a mirror modulation module [265], capable of simultaneously measuring long in-plane displacements (hundreds of millimeters) and short out-of-plane displacements (hundreds of micrometers). The system achieved a displacement resolution of 1.63 nm in the X-direction and 0.75 nm in the Z-direction. Preliminary accuracy tests indicated that the standard deviation for in-plane measurements was 6.37 nm, while for out-of-plane measurements, it was 3.69 nm. The heterodyne grating interferometers proposed by Zhu and Wang, as discussed earlier [172,208], both utilized multi-mirror modules for diffraction light modulation.

The diffraction light modulation modules discussed above primarily focus on collimating the diffracted beams from the gratings. However, considering the beam diameter and the size of the modulation module, this method is highly sensitive to working distance. Changes in working distance can directly cause the diffracted beams from the measurement and reference targets to fail to interfere or cause the diffracted beam from the measurement target to move out of the modulation module’s working range [243,244,267]. To address this issue, some researchers have proposed the Littrow modulation module [268,269]. In this structure, the incident beam enters the grating surface at a specific angle (typically the grating’s diffraction angle, also known as the Littrow angle). After diffraction by the grating, the positive and negative first-order diffracted beams return along the reverse path of the incident beam. In this configuration, the grating serves as both a diffractive and reflective element, eliminating the need for additional mirrors [214,270]. This makes the system more compact and less sensitive to the working distance, greatly expanding the measurement range of grating measurement systems. 

In 2017, Tan et al. proposed a wide-range, three-axis grating measurement system with nanometer resolution [165], as shown in Figure 9g, based on a Littrow configuration, to extend the Z-direction measurement range. In this system, the ±1st-order diffracted beams from two planar gratings in the X and Y directions propagate back along their original incident paths, acting as self-collimating diffracted beams. As a result, the proposed system significantly enhanced the Z-axis measurement range. The Z-direction movement of the moving target surface did not affect the diffraction spots in the X and Y directions, and the system resolution reached 4 nm. Later, the same team used a corner cube prism to modulate the diffracted light [271], proposing a Littrow-based, multi-DOF measurement reading head for 6-DOF measurements. The overall reading head size was 69 mm × 51 mm × 41 mm. The experimental results showed a 30 s stability of 2.5 nm, and the periodic nonlinear errors in both measurement directions were smaller than the resolution (0.25 nm for in-plane motion and 0.15 nm for out-of-plane motion). However, multiple reading heads typically produce synchronization and crosstalk errors during operation. In 2021, Li et al. proposed a symmetric heterodyne grating displacement measurement method based on a 2-D grating and single-diffraction quadruple subdivision [250], achieving a Littrow effect through steering optics. The experimental results indicated that the system achieved a measurement resolution of better than 3 nm in the X and Y directions, with grating displacement measurement errors better than ±30 nm and ±40 nm over a 10 mm range, and repeatability errors better than ±25 nm. In 2022, the same research team further proposed a three-dimensional displacement measurement method based on 2-D gratings with double-channel Littrow equal-optical-path incidence to measure the three-dimensional displacements in the X, Y, and Z directions of a two-dimensional grating [266]. By combining 2-D gratings with the Littrow incidence method and rotating elements, the Littrow diffracted light of frequency f_1_ was made to interfere with the reference light of frequency f_2_. A separation-double-channel phase decoupling algorithm was used to obtain displacement data in the X, Y, and Z directions. The experimental results showed that within a 10 mm measurement range in the X, Y, and Z directions, all linear errors were within ±60 nm, and the test resolution was within ±5 nm. The Littrow-based structural design is highly significant for extending Z-direction displacement measurement. However, this method imposes stringent requirements on the Littrow modulation module, necessitating customization for different grating structures. The high manufacturing costs of the optical components and the demanding installation process are also significant considerations.

Some researchers have opted to use transmission gratings to modulate diffracted light. In this approach, a transmission grating is used to diffract the incident beam initially, splitting a single incident beam into multiple diffracted beams. When these diffracted beams reflect back into the optical path, the transmission grating also acts as a collimating device, making the system more compact. In 2012, Kimura et al. utilized a unidirectional transmission grating structure to propose a three-axis optical reading head with sub-nanometer resolution for platform motion measurement [89], as shown in Figure 9e. The dimensions of this three-axis optical reading head were 50 mm (X) × 70 mm (Y) × 40 mm (Z), as shown in Figure 9c. Testing demonstrated that the resolution in all three axes was better than 1 nm, with the peak-to-valley amplitude of interpolation errors being ±10 nm, ±10 nm, and ±3 nm in the X, Y, and Z directions, respectively. Additionally, the tolerance range for changes in the working distance in the Z direction was ±150 nm. Subsequently, Shimizu et al. improved the three-axis reading head by incorporating a mosaic grating [272], as shown in Figure 9d. The experiments validated the feasibility of the designed four-reading head optical sensor structure and the concept of the mosaic grating, extending the measurement range of the XY main axes.

Although grating interferometers offer significant advantages in achieving multi-DOF measurements, when a single grating interferometer is used to measure the six degrees of freedom of a spatial object, the coupling error model between different degrees of freedom becomes highly complex. Achieving a high-precision displacement resolution remains a major challenge that needs to be addressed.

#### 3.4.3. Interferometric Optical Path Integration Module

In grating measurement systems, the interferometric optical path plays a crucial role in adjusting the polarization state of the laser beams and combining the beams [273]. The primary function of the interferometric optical path integration module is to integrate the entire measurement optical path, making it compact while ensuring that the measurement beam and the reference beam can interfere, thereby enhancing the stability and anti-interference capabilities of the entire measurement system. Some researchers have conducted in-depth studies on the integration of interferometric optical path modules.

The most typical module is the polarization state conversion module, which consists of a polarizing beam splitter (PBS) and two linear polarizers [237,243]. The fast axes of the two linear polarizers are oriented at 45 degrees relative to the horizontal axis of the PBS. This setup converts the polarization states of the reference and measurement beams so that after reflecting back into the diffraction optical path and combining, they share the same polarization state, fulfilling the interference condition necessary for interference to occur. This configuration is widely used in grating measurement systems [244,260]. Some researchers prefer integrating specific interferometric optical paths. In 2019, Ye et al. integrated the interferometric optical path module into an encoder reading head [261], as shown in Figure 9i, utilizing a fiber-free coupler to receive high-contrast interference beam signals under large-angle displacement conditions and generating a reference beam within the encoder head to suppress the thermal drift of the interferometer. The experimental results showed that the designed grating interferometer could achieve sub-nanometer displacement measurement in both in-plane and out-of-plane directions within 30 s, with 3σ values of 0.246 nm and 0.465 nm, respectively. The integrated interferometric optical path module facilitates integration and installation while reducing the sensitivity of optical components to environmental temperature changes. In 2022, Yin et al. proposed a high-precision, 2-D grating displacement measurement system based on dual spatial heterodyne optical paths arranged in an interleaved structure [251], as shown in Figure 9h. The system utilizes Doppler-controlled interferometry, heterodyne interferometry, and a dual spatial unit interleaved structure based on a 2-D grating to achieve the high integration of the entire interferometric optical path module, decoupling the optical phase changes caused by the 2-D grating and the interference signals. The experimental results demonstrated a resolution of 3 nm, with measurement errors in the 2-D grating direction better than ±175 nm and ±150 nm within a 40 mm range. Further optimization and testing of the design showed that within a 40 mm range, the error in the X direction was better than +50 nm and −40 nm, and in the Y direction, it was better than +100 nm and −80 nm.

Integrating the interferometric optical path module significantly enhances the overall stability of the measurement system and reduces the interference of environmental noise, facilitating the commercialization of the measurement system. However, customized optical modules lack universality and require high precision in the manufacturing process of optical components, leading to higher costs for the overall measurement system.

#### 3.4.4. Signal Processing Systems

The back-end signal processing system of a grating measurement system is crucial in industrial applications as it directly affects measurement accuracy and system stability. It requires high precision, stability, and strong anti-interference capabilities [274,275,276]. With the continuous development of programmable logic devices, particularly field-programmable gate arrays (FPGAs), these systems have gained significant flexibility and programmability [169,277]. Some newer chips have also integrated embedded technologies, enabling them to perform more complex computations and allowing for more flexible programming methods. Moreover, they can handle real-time signal processing [278,279]. This advancement is vital for enhancing the performance of grating measurement systems in dynamic and challenging industrial environments.

Many researchers have utilized FPGAs to develop signal processing systems for grating measurements [280,281]. In 2011, Jamro et al. combined the high sampling frequency of FPGAs with the flexibility of ARM microprocessors to propose a heterogeneous FPGA-ARM system capable of sampling and processing signals at a rate of 10 MS/s across individual channels [282]. In 2012, the PTB laboratory in Germany designed a laser interferometry signal processing platform based on the XILINX XC3S1000 series FPGA [262], as shown in Figure 9j. This platform integrated a 100 M high-speed, analog-to-digital converter and implemented algorithms such as phase-locked amplification, quadrature signal subdivision calculation, and Kalman filtering directly on the FPGA board. It could process signals with frequencies ranging from 10 kHz to 20 MHz, achieving a maximum measurement error of less than 75 pm during long-term measurements of commercial interferometers. In 2019, Li et al. proposed a real-time data processing hardware system for multi-axis grating interferometers that supported high-precision measurements of 2-D gratings [283]. This system could output measurement results at a rate of 2500 points per second at a motion speed of 1 m/s, with a measurement accuracy of better than 50 nm. In 2020, Han et al. developed a new generation grating interferometer data processing system based on an FPGA platform capable of simultaneously processing eight channels of interferometric signals [164], as shown in Figure 9k. Compared to traditional MATLAB-based offline data processing methods, the real-time processing error on the FPGA platform was less than 0.006 μm. Following this, Shi et al. developed an FPGA platform capable of simultaneously processing 40 signal channels [284,285], enabling the high-precision, real-time measurement of six-degree-of-freedom motion errors. The system achieved a linear displacement error of less than 1 nm and an angular displacement error of less than 0.5 arcseconds under a travel range of 18 mm and a rotation frequency of 0.5 Hz, with a system delay of less than 15 ms. In 2022, Wang et al. applied a nonlinear Kalman filter to grating interferometer signal processing, achieving simultaneous filtering, amplitude normalization [252], DC bias decoupling, harmonic suppression, and phase compensation. This significantly reduced the computational load and improved the real-time performance, with a single-channel output delay of only 1.8 μs at a 50 MHz clock frequency and a measurement error of less than 5 μm.

Despite significant advances in real-time processing systems, there are currently no industrialized modules for real-time processing. The development of such systems involves substantial hardware knowledge and high development costs. Particularly under high-channel-count and high-precision requirements, the complex logic design and debugging process can extend the system development cycle [286]. The performance of hardware systems heavily depends on the optimization of filter parameters and hardware configurations. If parameter settings are inaccurate or hardware resources are insufficient, it could affect the measurement accuracy and the system stability [287,288]. Although some integrable grating interferometer components are available, most are still custom-made optical devices tailored to specific grating interferometer systems. This lack of generalization severely hinders the industrialization and broader development of grating interferometers.

Table 2 shows the main development directions, current status, and limitations of advanced grating interferometry technology.

## 4. Gratings in Miniature Spectrometers

**Figure 10:**
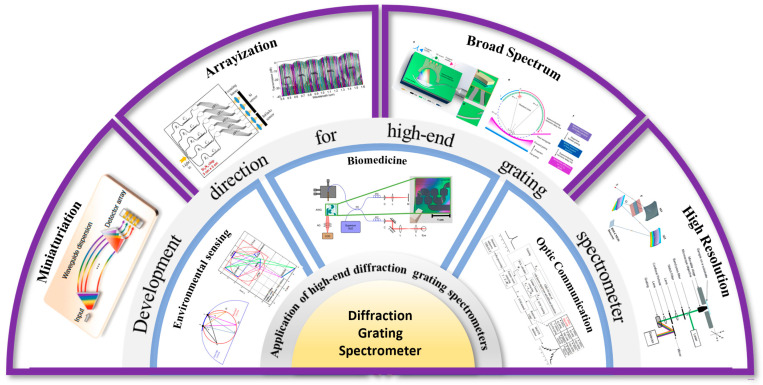
Application and development direction of grating spectrometer [74,289,292,293,294,295,300,301].

Spectrometers are extensively utilized in various fields, such as biomedical science [289,290,291], environmental sensing [292], and optical communication [293], as shown in Figure 10 Ideally, these instruments should offer a broad spectral range [74,294,295], arrayed test capability, small size [296], cost-effectiveness [297], and high resolution [298,299]. Gratings are the core components of dispersive spectrometers [10]. The miniaturization of spectrometers primarily involves integrating multiple optical functions into a single element to replace traditional components. In conventional spectrometer systems, the core functionalities of focusing and dispersion can be combined using diffractive structures on the surfaces of refractive or reflective optical elements, such as concave gratings and Fresnel gratings, which serve as hybrid components. These spectrometers typically require minimal optical elements to achieve the full functionality of the instrument.

### 4.1. Concave Grating Spectrometer

**Figure 11:**
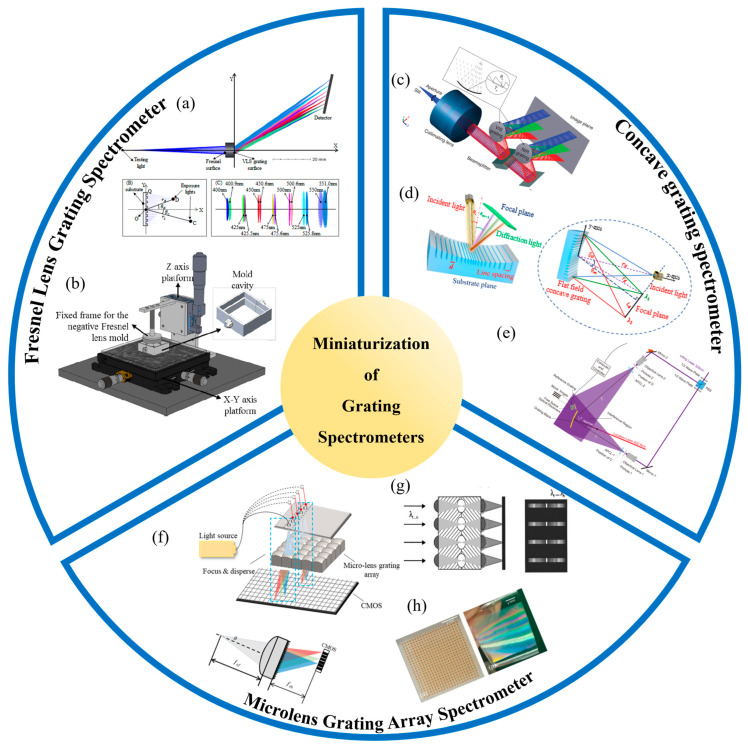
Miniaturization of grating spectrometers: (**a**) a schematic of the simulated Fresnel spectrometer system with ZEMAX [304]; (**b**) a schematic of the exposure system for Fresnel grating fabrication [305]; (**c**) a two-channel broadband spectrometer that employs variable-spacing concave blazed gratings [47]; (**d**) a schematic of multiwavelength confocal displacement sensing using high dispersion flat-field concave gratings [145]; (**e**) a schematic diagram of a multivariable line space concave grating with high diffraction efficiency [303]; (**f**) a schematic diagram of a microlens array grating for multi-channel spectrometers [306]; (**g**) a schematic of a micro-spectrometer array created using a holographic recording [151] grating directly on a microlens; (**h**) a grating/microlens array for micro-spectrometers, fabricated using hot-melt, self-assembly, and replication techniques [153].

Recent advancements in miniaturized spectrometer design have leveraged concave gratings to achieve compact, high-performance optical systems [96,302]. Here are some key studies that illustrate distinct approaches to optimize these systems for various applications. Figure 11c describes a two-channel broadband spectrometer that employs variable-spacing concave blazed gratings [47]. This design achieves a spectral resolution of 0.1 nm over a broad wavelength range of 400–1100 nm, all within a compact form factor. The dual-channel configuration, with independent gratings tailored for different spectral regions, enhances both sensitivity and versatility, making this system highly adaptable for applications demanding wide spectral coverage and high precision. Figure 11d focuses on multi-wavelength confocal displacement sensing, leveraging a highly dispersive flat-field concave grating [145]. This system achieves sub-micron displacement resolution with a measurement range of up to 10 mm across multiple wavelengths (450–700 nm). The innovative use of a single concave grating to disperse and focus light onto the detector simplifies the optical layout, reducing system complexity and size, which is critical for precision sensing in space-constrained environments. Figure 11e addresses the challenges of ultraviolet (UV) spectroscopy in astronomy, particularly within the Lyman UV range (90–120 nm) [303]. By developing high diffraction efficiency varied-line-space concave gratings, this research achieves a spectral resolution of 0.05 nm and a diffraction efficiency exceeding 30% across the target UV range. The precision manufacturing of these gratings ensures minimal aberrations, providing a robust solution for high-resolution UV spectrometry, essential for advanced astronomical observations.

Collectively, these studies highlight the versatility and effectiveness of concave gratings in advancing miniaturized optical systems. They demonstrate how sophisticated grating designs can push the boundaries of spectral resolution, sensitivity, and efficiency, paving the way for next-generation spectrometers that meet the rigorous demands of modern scientific and industrial applications. Furthermore, Zhou. and Li. have investigated the fabrication processes of concave gratings (as shown in Section 2.4). However, due to the low success rate of creating specific grating structures on curved surfaces, their large-scale production and application have been challenging.

### 4.2. Fresnel Grating Spectrometer

To address the challenges associated with the fabrication of flat-field, concave gratings [132,133,134,135,136], a composite structure that combines lenses with gratings—known as the Fresnel grating—was proposed by the University of Pennsylvania in 2010 [307]. A Fresnel grating is a planar optical element with one side designed as a Fresnel lens and the other as a grating. Unlike flat-field concave gratings, which require modifications to the groove profile for collimation, Fresnel gratings achieve collimation and focusing by utilizing a lens configuration. Liu et al. developed a transmission-type Fresnel grating miniature spectrometer suitable for smartphones [308]. Additionally, Geng et al. designed and fabricated a variable-line-space reflective Fresnel grating. As illustrated in Figure 11a [304], the designed optical path allows for the adjustment of the grating’s line spacing, enabling its adaptation to various spectral ranges and resolutions, thereby optimizing the performance of the spectrometer.

The manufacturing process for Fresnel gratings involves inserting a soft PDMS layer between a hard grating and a pre-copied negative Fresnel surface, which facilitates the simultaneous creation of both the Fresnel and grating surfaces. Several techniques aimed at reducing adhesion were also explored, contributing to enhanced manufacturing efficiency and cost-effectiveness by minimizing curing time and prolonging mold lifespan. A compact fabrication platform was established for producing a G-Fresnel lens with a diameter of 25.4 mm, an equivalent focal length of 25 mm, and a grating pattern with 600 grooves/mm, as depicted in Figure 11b [305]. This platform occupies a volume of less than 160 × 140 × 106 mm³, allowing it to fit within a standard vacuum drying oven. Furthermore, a prototype spectrometer utilizing the G-Fresnel lens was constructed and evaluated. This spectrometer boasts a compact size of approximately 100 mm × 50 mm × 30 mm and operates over a wide wavelength range from 450 nm to 650 nm. The results confirm that the spectrometer equipped with the G-Fresnel lens can achieve spectral resolutions superior to 1.2 nm.

### 4.3. Novel Microlens Grating Spectrometer

Moreover, innovative research has led to the development of multi-channel spectrometers. This approach has the potential to address key challenges in miniaturization while maintaining high spectral resolution and efficiency. Figure 11f introduces a microlens array grating specifically designed for multi-channel spectrometers [306]. This design employs a two-dimensional microlens array that acts as a focusing element, directing light onto a planar diffraction grating. The system is capable of resolving multiple spectral channels simultaneously, with each microlens directing light from a specific channel onto the grating. The array configuration allows for a high degree of parallelism in spectral data acquisition, making the spectrometer highly efficient in terms of data throughput. The compactness of the system is achieved without compromising spectral resolution, which is maintained at around 1 nm across a wavelength range of 400–700 nm. This approach effectively combines the benefits of microlenses and diffraction gratings, resulting in a miniaturized spectrometer that is well-suited for applications requiring rapid, multi-channel spectral analyses.

The study, shown in Figure 11g [151], by Traut explores the use of holographically recorded gratings directly on microlenses to create a miniaturized spectrometer array. The innovative aspect of this study lies in the integration of the grating within the microlens itself, eliminating the need for separate optical components. This integration simplifies the optical path and reduces the overall size of the spectrometer. The holographic recording process allows for precise control over the grating parameters, resulting in high diffraction efficiency and spectral resolution. The system achieves a resolution of 0.5 nm over a wavelength range of 450–850 nm, demonstrating the potential of this technique for creating highly compact and efficient spectrometers. The direct integration of the grating and microlens also enhances the system’s alignment stability, a critical factor in maintaining performance in miniaturized optical devices. The study shown in Figure 11h presents a novel method for fabricating grating/microlens arrays using hot-melting, self-assembly, and replication techniques [153]. This approach addresses the challenges of large-scale production and cost-effectiveness in miniaturized spectrometer design. By utilizing self-assembly, the microlens array and grating are fabricated simultaneously, ensuring precise alignment and consistency across the array. The replication process enables the production of multiple identical units, making this method highly scalable. The resulting spectrometer achieves a spectral resolution of approximately 2 nm across a wavelength range of 350–750 nm, with a compact design that is suitable for integration into portable devices. The use of hot-melting techniques also improves the durability of the optical components, making the system more robust for practical applications.

In summary, these studies demonstrate significant advancements in the integration of microlens arrays with gratings for miniaturized multi-channel spectrometers. Each study offers a unique approach to addressing the challenges of miniaturization, from the use of microlens arrays for parallel spectral data acquisition to the holographic integration of gratings and the development of scalable fabrication techniques.

## 5. Conclusions and Prospect

This review presents an overview of recent advancements in holographic interferometric fabrication, grating interferometry, and spectrometer technology, highlighting their critical roles in precision measurement and modern optical systems. The development of these technologies, particularly laser interference lithography (LIL) for grating fabrication, has significantly contributed to high-resolution spectral analysis and other optical applications. Additionally, grating interferometry has achieved remarkable progress in achieving nanoscale and sub-nanoscale measurement accuracies, enabling the development of advanced multi-degree-of-freedom, high-precision measurement systems.

In the field of interference lithography, LIL has emerged as a core technique for grating manufacturing. Innovations such as multi-beam interference, fringe-locking systems, and advanced polarization control have markedly enhanced the precision and reliability of gratings, broadening their application scope and facilitating advancements in spectrometer technology. Grating interferometry, in homodyne and heterodyne measurement systems, has reached unprecedented levels of measurement accuracy. While the homodyne grating interferometer offers a nanometer-level measurement accuracy and has a relatively compact structure, it still faces issues with a weak signal anti-interference capability and signal drift. In contrast, the heterodyne grating interferometer, though capable of achieving sub-nanometer precision, has a very large measurement system, is structurally complex, and incurs high costs. Grating interferometers currently encounter significant challenges in industrial applications, mainly due to problems with the system module integration, anti-interference capacity, and long-term stability. Meanwhile, advancements in spectrometer technology have been driven by the development of innovative integrated structures, such as concave gratings, Fresnel gratings, and grating–microlens arrays, which are central to the miniaturization of spectrometers, thereby expanding their potential use in compact analytical instruments.

Future research should focus on optimizing the fabrication processes for complex grating structures, exploring new materials and techniques to achieve even higher precision, and expanding the use of grating technologies into emerging fields, like biomedical imaging, environmental monitoring, and quantum computing. Overcoming the current limitations in grating interferometry, particularly in terms of the system integration and anti-interference capabilities, will be essential for broader industrial applications. Addressing these challenges will unlock the full potential of grating technologies, furthering their impact on both scientific research and industrial applications.

## Figures and Tables

**Table 1:** Main micro–nano fabrication technology behind grating fabrication.

Grating Fabrication Tech	Advantages	Disadvantages
Mechanical Ruling	With the development of Fast Tool Servo (FTS) technology, complex pattern structures can be machined.	Tool wear and high running accuracy, minimum encoder pitch is limited by the tool (typically up to a few micrometers).
Nanoimprint Lithography	High resolution for small cycle grating fabrication; high productivity.	The cost of the equipment is high, and the precision of the mask is required.
Projection Exposure	Higher productivity and resolution.	High equipment and mask cost.
Electron Beam Lithography (EBL)	High resolution for grating fabrication of small apertures (less than 100 nm).	High costs, only small areas can be processed (no more than 100 × 100 µm^2^).
Laser Interference Lithography (LIL)	Sub-micron periodic grating fabrication at high efficiency and low cost.	Has difficulty processing complex structural patterns.

**Table 2:** Grating interferometry technology: current development status and limitations.

Main Development Directions for Advanced Grating Interferometry Technology	Development Status	Developmental Limitations
Ultra-high-precision measurement	Sub-nanometer-level resolution Sub-nanometer-level accuracy	Complex structure Lack of stability
Absolute measurement	Nanometer-level resolution Nanometer-level accuracy	Absolute encoding design Lack of ultra-high accuracy
Multi-DOF measurement	Expandable to 6-DOF measurement	Complex structure
Industrialization development	Partially industrializable modules	Lack of mature modules

## Data Availability

The data presented in this study are available on request from the corresponding author.
